# Impact of close interpersonal contact on COVID-19 incidence: Evidence from 1 year of mobile device data

**DOI:** 10.1126/sciadv.abi5499

**Published:** 2022-01-07

**Authors:** Forrest W. Crawford, Sydney A. Jones, Matthew Cartter, Samantha G. Dean, Joshua L. Warren, Zehang Richard Li, Jacqueline Barbieri, Jared Campbell, Patrick Kenney, Thomas Valleau, Olga Morozova

**Affiliations:** 1 Department of Biostatistics, Yale School of Public Health, New Haven, CT, USA.; 2 Department of Statistics and Data Science, Yale University, New Haven, CT, USA.; 3 Department of Ecology and Evolutionary Biology, Yale University, New Haven, CT, USA.; 4 Yale School of Management, New Haven, CT, USA.; 5 Epidemic Intelligence Service, Centers for Disease Control and Prevention, Atlanta, GA, USA.; 6 Infectious Diseases Section, Connecticut Department of Public Health, Hartford, CT, USA.; 7 Department of Statistics, University of California, Santa Cruz, Santa Cruz, CA, USA.; 8 Whitespace Ltd., Alexandria, VA, USA.; 9 Program in Public Health and Department of Family, Population and Preventive Medicine, Stony Brook University, Stony Brook, NY, USA.

## Abstract

Close contact between people is the primary route for transmission of SARS-CoV-2, the virus that causes coronavirus disease 2019 (COVID-19). We quantified interpersonal contact at the population level using mobile device geolocation data. We computed the frequency of contact (within 6 feet) between people in Connecticut during February 2020 to January 2021 and aggregated counts of contact events by area of residence. When incorporated into a SEIR-type model of COVID-19 transmission, the contact rate accurately predicted COVID-19 cases in Connecticut towns. Contact in Connecticut explains the initial wave of infections during March to April, the drop in cases during June to August, local outbreaks during August to September, broad statewide resurgence during September to December, and decline in January 2021. The transmission model fits COVID-19 transmission dynamics better using the contact rate than other mobility metrics. Contact rate data can help guide social distancing and testing resource allocation.

## Teaser

Close interpersonal contact measured using mobile device location data explains dynamics of COVID-19 transmission in Connecticut.

## INTRODUCTION

Close contact between people is the primary route for transmission of the novel severe acute respiratory syndrome coronavirus 2 (SARS-CoV-2), the virus that causes coronavirus disease 2019 (COVID-19) (*1*). Social distancing guidelines published by the U.S. Centers for Disease Control and Prevention (CDC) recommend that people stay at least 6 feet away from others to avoid transmission via direct contact or exposure to respiratory droplets (*2*). Throughout the world, nonpharmaceutical interventions, including social distancing guidelines and stay-at-home orders, have been used to encourage the physical separation of people and reduce the risk of COVID-19 transmission via close contact (*3*–*6*). U.S. states with the lowest levels of self-reported social distancing behavior are experiencing most severe COVID-19 outbreaks (*7*).

While individual-level compliance with social distancing guidelines can be difficult to measure, researchers have proposed population-level mobility metrics based on mobile device geolocation data as a proxy measure for physical distancing and movement patterns during the COVID-19 pandemic (*8*–*12*). Investigators have characterized geographic and temporal changes in mobility metrics following nonpharmaceutical interventions such as social distancing guidelines and stay-at-home mandates during the COVID-19 pandemic (*11*, *13*–*16*). Researchers have also studied the association between mobility metrics and COVID-19 cases or other proxy measures of transmission (*11*, *17*–*28*). Most mobility metrics measure aggregated movement patterns of individual mobile devices: time spent away from home, distance traveled, or density of devices appearing in an area during a given time interval. CDC reports mobility metrics from Google, Safegraph, and Cuebiq (*29*). Some mobility metrics measure spatial relationships among individual devices. Klein *et al.* (*12*) measure “colocation” events in which reported locations of two devices lie within a roughly 60–square foot spatial grid cell. Couture *et al.* (*30*) compute a “device exposure index” that measures the colocation of devices within a sample of preselected venues such as restaurants or retail establishments. Chang *et al.* (*24*) use colocation matrices from Facebook (*31*) that measure the probability that devices from different geographic areas appear in the same 600-m^2^ region for 5 min, aggregated by week. Morley *et al.* (*32*) use the “human encounters” metric from Unacast (*33*, *34*) that measures the frequency of two devices being within 50 m of each other for an hour or less. Last, Cuebiq offers a contact index measuring when two or more devices are within 50 feet of each other within 5 min (*35*).

Existing mobility metrics might not capture simultaneous colocation of devices, do not measure contact within a 2-m distance associated with highest transmission risk (via direct contact or exposure to respiratory droplets), and might not take intrinsic mobile device spatial location error (horizontal uncertainty) into account. A better measure of contact events, the primary behavioral risk factor for transmission, could help explain historical patterns of transmission, assist policymakers in targeting interventions and messaging campaigns to encourage social distancing, guide public health response measures such as enhanced testing and contact tracing, and provide early warning to detect and prevent emerging outbreaks. By using highly detailed mobile device geolocation data and a novel probabilistic method for assessing close proximity, we sought to quantify total intensity of close interpersonal contact (within 6 feet) at the population-level (contact rate) and to use contact rate to explain patterns of COVID-19 incidence and predict emergence of new COVID-19 cases in the state of Connecticut, USA during 1 February 2020 to 31 January 2021.

### Setting: Connecticut

Connecticut (population, 3.565 million), similar to other states in the northeastern United States, experienced a strong initial wave of COVID-19 infections during March to April 2020 following outbreaks in the New York City area (*36*, *37*). On 17 March, Connecticut Governor Ned Lamont closed schools and issued a statewide “Stay Safe, Stay Home” mandate to take effect on 23 March 2020. Governor Lamont’s executive order recommended that nonessential businesses cease all in-person functions, closed in-person dining at restaurants, and canceled all in-person community gatherings. The mandate excluded health care, food service, law enforcement, and other essential services.

As case counts declined, Connecticut followed a gradual reopening plan designed to resume economic activity while minimizing the risk of transmission via close contact between people. On 20 May, the state entered phase 1, permitting the following to open at 50% capacity with social distancing: hair salons and barbershops, outdoor zoos and museums, outdoor dining, outdoor recreation, retail shopping, university research, and offices, although work from home was strongly encouraged. On 17 June, phase 2 began, permitting indoor religious services at 25% capacity and capped at 100 people; outdoor religious services capped at 150 people; and opening indoor dining, hair salons, personal service businesses, and libraries at 50% capacity. A serology study to measure prevalence of SARS-CoV-2–specific immunoglobulin G antibodies was conducted among adult Connecticut residents residing in noncongregate settings during June to July (*38*). The study estimated a seroprevalence of 4.0% (90% confidence interval, 2.0 to 6.0%). Participants in the study reported their risk mitigation behaviors: 73% avoided public places, 75% avoided gatherings of families or friends, and 97% wore a mask at least some of the time. In July, Governor Lamont delayed the state’s planned summer move to phase 3, which would have loosened occupancy restrictions on bars and restaurants, because of surges in transmission occurring elsewhere in the United States.

Connecticut experienced low COVID-19 incidence and declining hospitalization during June to August, but in August, a major outbreak occurred in Danbury, a town in the western part of the state (*39*). During August to September, in-person education resumed at many colleges, universities, and primary/secondary schools in Connecticut. By mid-September, the state was facing a broad resurgence of COVID-19 transmission. On 17 September, the Connecticut Department of Public Health reported that the number of new cases per week for the previous 4 weeks was 62% higher than the average number of new cases per week in July and early August (*40*). These signs of resurgence were initially concentrated in southeastern Connecticut, where few COVID-19 cases were identified during the initial spring wave in March to April (*41*, *42*).

Public health officials identified travel, social gatherings, workplaces, churches, universities, and recreational sports as contributing to transmission. Nevertheless, on 8 October, the state began phase 3 reopening, permitting 50% capacity in houses of worship capped at 200 people and uncapped outdoor religious gatherings with social distancing and opening indoor dining, hair salons, personal service businesses, and libraries at 75% capacity. On 6 November, as COVID-19 case counts continued to increase, Connecticut reverted to “phase 2.1”, reducing indoor restaurant seating, indoor and outdoor event capacity, and placing caps on attendance. Case counts increased through December and began to decline in January 2021. Overall, Connecticut residents complied with state guidelines and mandates to reduce close contact. In a survey of risk mitigation behaviors throughout the United States, Lazer *et al.* (*7*) reported that Connecticut ranked ninth among U.S. states in self-reported social distancing during fall 2020 and sixth in self-reported mask wearing. However, case counts indicate that Connecticut experienced widely varying temporal and geographic dynamics of COVID-19 incidence over the course of the pandemic.

## MATERIALS AND METHODS

We obtained raw mobile device geolocation data for a sample of devices in Connecticut from X-Mode, a company that provides mobile device location data. X-Mode provides software development kits to application developers, collects device location data from the applications, and licenses location data to customers. Through a licensing agreement between X-Mode and Whitespace Ltd., the authors purchased device location data from X-Mode under a contract with the Connecticut Department of Public Health to provide contact metrics for COVID-19 risk monitoring. Location data used in this work is collected from devices via third-party applications. Users have agreed via application terms and consent forms to collection of location information. Location data may be collected even when users are not interacting with the applications that report data. A combination of factors—device operating system, application usage, and user behavior—all affect the frequency of reporting.

During 1 May 2020 through 31 January 2021, we observed a total of 788,842 unique device IDs, representing roughly 22% of the approximately 3.565 million residents of Connecticut (although some of those devices may have belonged to people residing elsewhere). An average of 141,617 unique devices were observed per day. For each week, an average of 80.5% of device IDs from the prior week were present in the data. Devices might not be present in the dataset if the user turns off the device or does not interact with applications that report location data. Devices report more GPS location data when they are in use and may not report at all when the device is not being used. We observe an average of 100 to 200 location points per day per device. Using device geolocation records consisting of device IDs, GPS coordinates, date/time stamps, and GPS location error estimates (horizontal uncertainty), we calculated the location in which each device had the most location records and designated that area as the device’s primary dwell location (i.e., town of residence of device owner). In the Supplementary Materials, we present a detailed analysis of unique device coverage over time, number of location points per device per day, and number of location points per minute averaged over days.

**Fig. 1.**
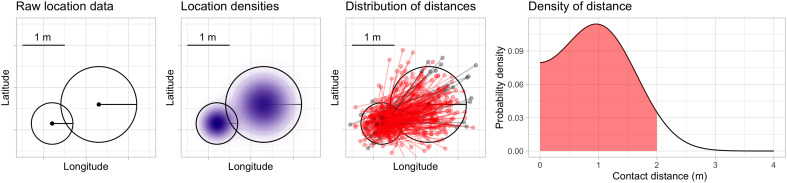
Schematic illustration of contact probability calculation. (**Left** to **right**): Raw locations, including horizontal uncertainty estimates, for two mobile devices are transformed into approximate location probability densities. The distribution of distances from points drawn randomly from these densities is computed. Sampled distances are shown here for illustrative purposes in red (when sampled device locations are within 6 feet apart) and gray (when sampled locations are more than 6 feet apart); in our implementation, the distribution of these distances is computed analytically. The shaded area under the density curve is the probability that the devices are within 6 feet.

A contact event was computed by using a probabilistic algorithm that computes the likelihood of simultaneous 2-m proximity between pairs of devices across geographic areas. For each device, we identify sets of records where devices were in spatial proximity to one another and stationary. A limitation of mobile device geolocation data is that it is not possible to precisely quantify the duration a device is stationary because device locations are collected asynchronously and irregularly over time. For each potential contact event, we compute the probability that the two device locations are within 6 feet by assuming that the reported device locations arise from a two-dimensional Gaussian probability distribution whose variance is computed by using the horizontal uncertainty measure and correct the distance to account for the curvature of the earth. Figure 1 shows a schematic illustration of the contact event probability calculation. We define the “contact rate” as the total number of contact events per day among observed devices at the town level; the contact rate is computed by summing daily contact probabilities for each device and assigning that sum to the device primary dwell location.

The mobile device location data contain no individually identifying metadata, but raw locations can reveal potentially identifying information about locations where the user spends time. We therefore use the raw location data to compute contact counts aggregated by day and town. These contact data are not individually identifiable. This work was approved as “not human subjects research” by the Yale University institutional review board. This work was also reviewed by CDC and was conducted consistently with applicable federal law and CDC policy. A detailed description of the mobile device geolocation data, computation of the probability of contact, spatial aggregation of the contact probabilities to estimate contact rate, and coverage of mobile devices across Connecticut is given in the Supplementary Materials.

## RESULTS

### Statewide contact trends

**Fig. 2.**
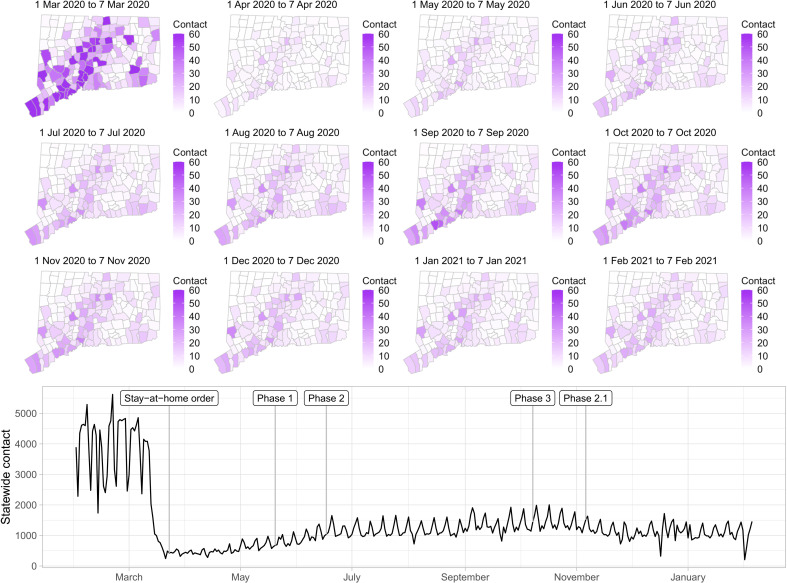
Estimated contact rate among mobile devices in our dataset in Connecticut from February 2020 to February 2021. (**Top**) Maps show the number of contacts in Connecticut’s 169 towns per day during weeks beginning on the first of each month. Darker colors indicate higher contact. (**Bottom**) Statewide contact shows the daily frequency of close contact within 6 feet between distinct devices in our dataset. Governor Ned Lamont’s stay-at-home order and reopening phases 1, 2, 3, and 2.1 are indicated. The state reverted to the more restrictive “phase 2.1” in response to rising case counts in November.

Figure 2 shows the contact rate by town in Connecticut during 1 February to 31 January 2021. Maps show the weekly average of daily contact rate by town, where darker colors in maps indicate higher contact rate. The daily contact rate is shown in the plot below. The statewide contact rate dropped markedly in March, about 1 week before Governor Lamont issued the statewide stay-at-home mandate on 23 March. News of surging COVID-19 hospitalization and responses in the New York area, closure of public schools, and anticipation of a possible stay-at-home order might have played a role in reducing contact before the mandate was announced. After staying low during most of April, the contact rate began to rise slowly throughout the state during June to August. Incidence of infection was likely much higher during the first wave than the second, but steadily increasing availability of SARS-CoV-2 testing yielded higher case counts in the second wave. An interactive web application for exploring the contact rate in Connecticut is available at https://forrestcrawford.shinyapps.io/ct_social_distancing. The Supplementary Materials describe the web application in detail.

The Supplementary Materials presents a comparison of the contact rate to mobility metrics from Google (*43*), Apple (*44*), Facebook (*45*), Descartes Labs (*13*, *46*), and Cuebiq (*35*). Most mobility metrics provided by these companies returned to values near the February/March baseline by the beginning of July. In contrast, the contact rate shown in Fig. 2 shows that close interpersonal contact stayed low and rose slowly during June to August 2020. Mobility metrics returned more quickly to the February 2020 baseline (or higher) compared to the contact rate and do not explain the low COVID-19 incidence achieved in Connecticut during June to August, 2020.

One explanation for the discrepancy between close contact and mobility metrics is that it is possible to travel far from home, to many distinct points of interest, or to many geographic areas, without coming into close contact with others. This might be what occurred in the summer of 2020: As Connecticut began its phased reopening plan, people resumed more normal patterns of away-from-home movement—work, shopping, or recreational activities—while maintaining social distancing. For this reason, when mobility metrics are used as proxy measures of close interpersonal contact, they might overstate the risk of disease transmission.

### Prediction of COVID-19 cases in Connecticut towns

To evaluate the contact rate as a predictor of COVID-19 burden in Connecticut, we predict transmission of SARS-CoV-2 and COVID-19 cases in Connecticut towns using a continuous-time deterministic compartmental transmission model based on the susceptible-exposed-infective-removed (SEIR) process (*47*). We accommodate geographical variation in transmission within Connecticut and estimated features of COVID-19 disease progression, hospitalization, and death. The model incorporates flexible time-varying case-finding rates (proportion of infections detected) at the town level. We incorporate the contact rate into the time-varying transmission risk by multiplying the standardized contact rate by the product of the baseline transmission rate and the estimated number of susceptible and infectious individuals in each town. We fit the model to statewide data and produce model projections for each of Connecticut’s 169 towns using the town population size, time-varying contact rate, estimated initial infection fraction, and time-varying case-finding rate. The model is conceptually similar to other SEIR-type COVID-19 transmission models making use of mobility data but incorporates much geographic variation in transmission rates (*20*, *48*–*56*). The model and calibration procedure are described in detail in (*47*) and in the Supplementary Materials.

We calibrate the model parameters at the state level using the observed dynamics of confirmed COVID-19 hospitalization census (number of patients currently hospitalized), cumulative COVID-19 hospitalizations, and cumulative number of deaths among hospitalized cases. We use confirmed COVID-19 case data from noncongregate settings reported to the Connecticut Department of Public Health. We excluded cases among residents of long-term care facilities, managed residential communities (e.g., assisted living facilities), or correctional institutions. We aggregated noncongregate case data by day of sample collection by town. We obtained town-level population estimates from the American Community Survey (*57*, *58*).

**Fig. 3.**
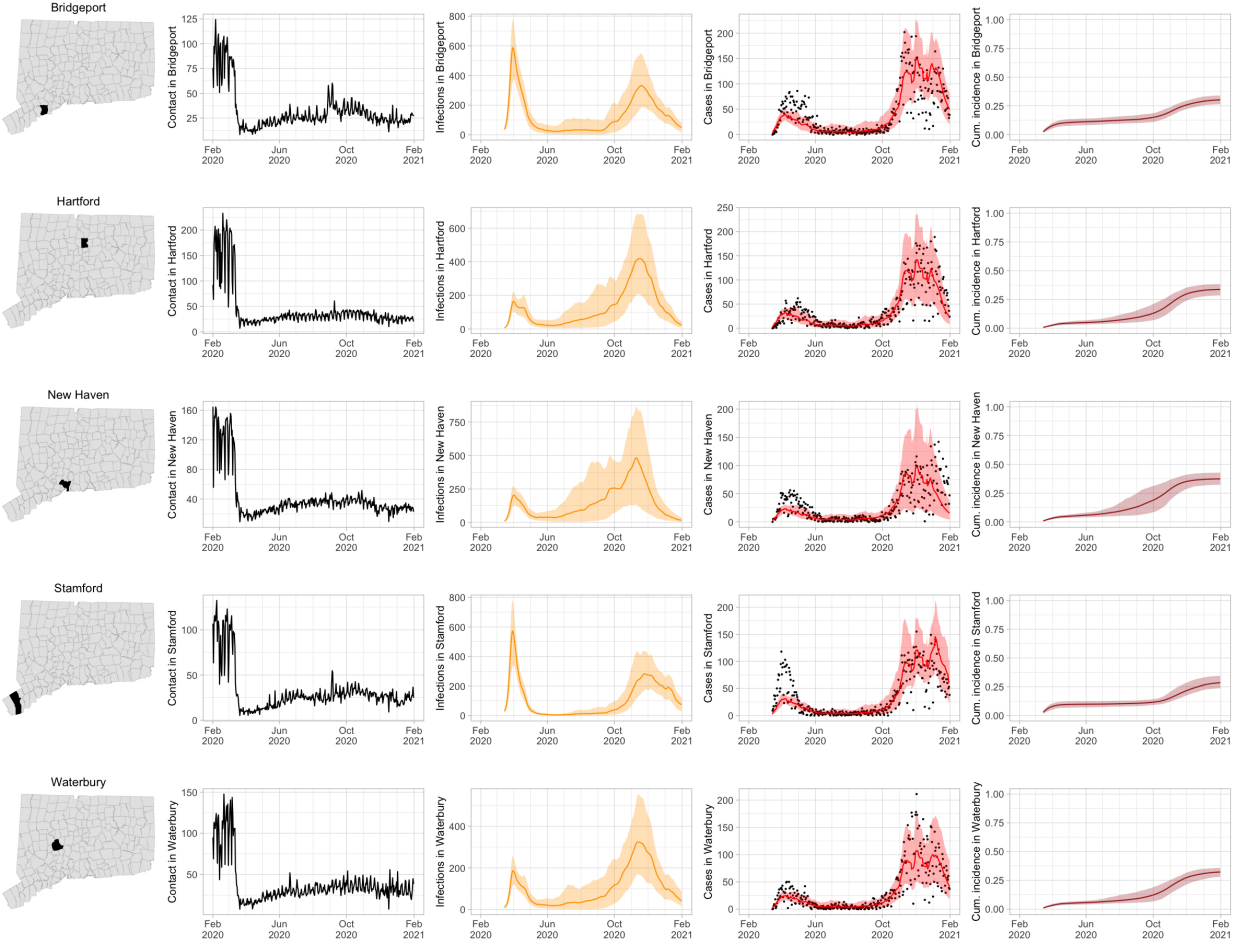
Illustration of contact rates (number of contacts per day) and number of COVID-19 cases and transmission model predictions (with 95% uncertainty intervals) of number of infections, cases, and cumulative incidence proportion (proportion of the population that has been infected) in the five largest cities by population in Connecticut: Bridgeport, Hartford, New Haven, Stamford, and Waterbury. Black dots show confirmed noncongregate COVID-19 case counts.

Figure 3 shows how contact rates are used to produce epidemiological inferences that follow the pattern of observed cases closely. Rows show contact rates and transmission model–predicted SARS-CoV-2 infections, observed and estimated case counts, estimated cumulative incidence (proportion of the population that has been infected), and 95% uncertainty intervals for model estimates for the five largest cities by population in Connecticut: Bridgeport, Hartford, New Haven, Stamford, and Waterbury. Contact rates in these towns largely mirror rates in the state as a whole. Model estimates track the pattern of case counts through the full course of the epidemic, including the marked reduction in transmission during June to August. In some towns, e.g., Stamford, case counts are underestimated in model projections during the first wave during March to April 2020. In these cases, dynamics of SARS-CoV-2 infections may differ from the dynamics of case counts because the estimated case detection rate (via viral testing) varied markedly over time and geography.

To evaluate the usefulness of the contact rate as an input to the epidemiological transmission model, we also fit the SEIR transmission model using mobility metrics from Apple, Descartes Labs, Facebook, and Google, as well as a no-mobility null model. The model including the contact rate fits best, and the estimated random effects function that accommodates variation in transmission not accounted for by contact is centered around zero. When no mobility metric is used, the estimated random effects function exhibits a large drop in spring/summer 2020, recapitulating the general pattern exhibited by the contact metric. Other mobility metrics exhibit poorer fit, although Descartes Labs mobility shows the best fit among the mobility metrics. A detailed comparison of model fit using the contact rate and alternative mobility metrics is shown in the Supplementary Materials.

### Role of contact in local outbreaks

**Fig. 4.**
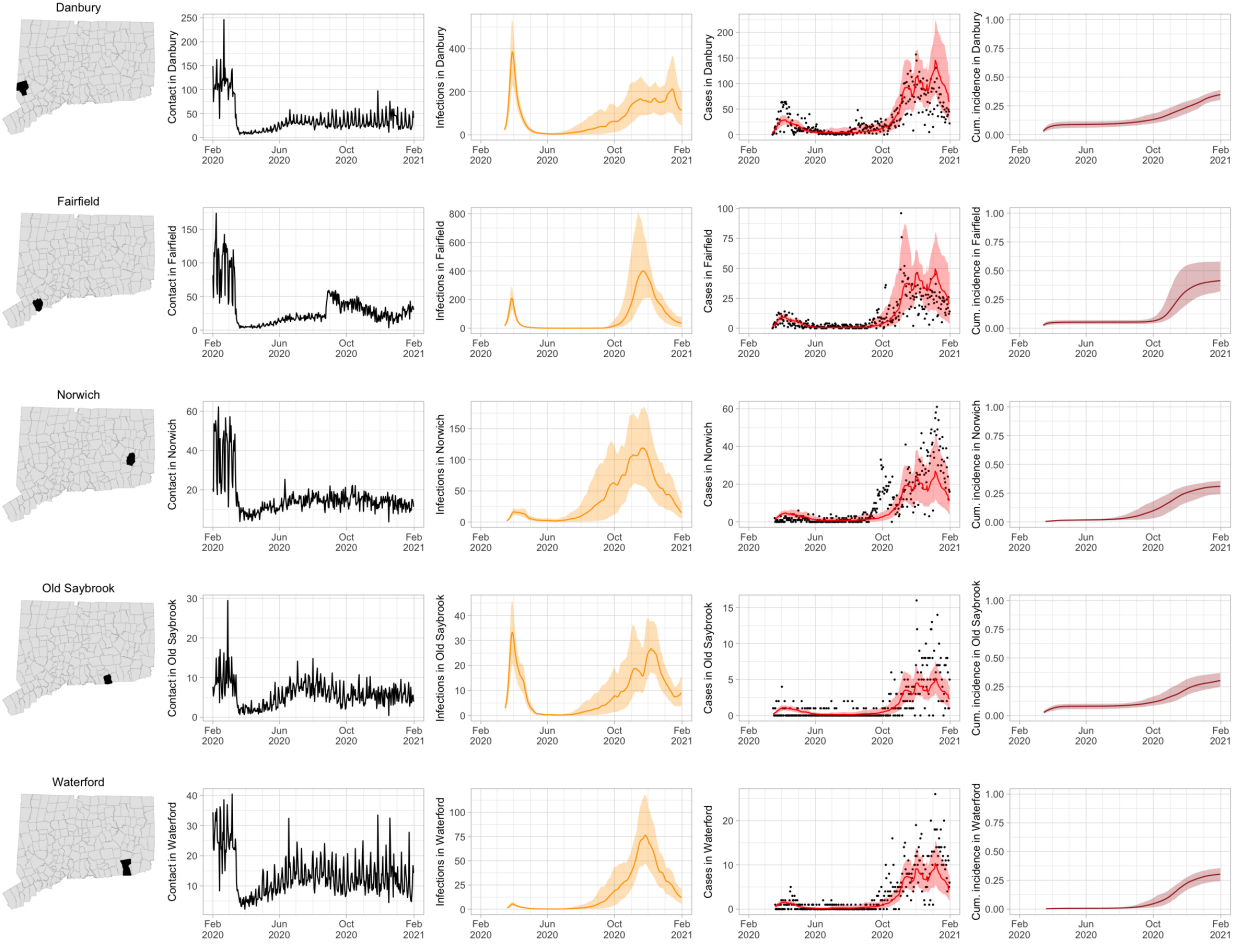
Illustration of contact rates (number of contacts per day) and number of COVID-19 cases and transmission model predictions (with 95% uncertainty intervals) of number of infections, cases, and cumulative incidence proportion (proportion of the population that has been infected) in several towns in Connecticut whose case or contact patterns differ from that of the state as a whole: Danbury, Fairfield, Norwich, Old Saybrook, and Waterford. Public health officials declared an outbreak in Danbury in mid-August 2020. Fairfield experienced outbreaks linked to two universities in September 2020. Norwich, Old Saybrook, and Waterford, in the eastern part of the state, were mostly spared during the first wave of infection and had quickly rising case counts in fall 2020.

As COVID-19 case counts in Connecticut decreased during June to August, new and more heterogeneous patterns of transmission emerged. Figure 4 shows contact rates, confirmed noncongregate COVID-19 case counts, and 95% uncertainty intervals for cases in five Connecticut towns where incidence patterns differed from those of the larger cities shown in Fig. 3.

During June to August, the only known community-wide COVID-19 outbreak in Connecticut occurred in the town of Danbury (population, 84,479) (*39*). During 2 to 20 August, at least 178 new COVID-19 cases were reported, an increase from 40 cases reported during the prior week. Contact tracing investigations by public health officials attributed the outbreak to travel, but the contact rate was high in Danbury beginning in July, and genomic analyses suggested that the outbreak was closely linked to lineages already circulating in New York City and Connecticut (*59*, *60*). Predictions from the model including contact rates from Danbury suggest that this outbreak might have been part of a long-term increase in infections that began earlier in July and continued mostly unabated through November.

The town of Fairfield, bordering the larger city of Bridgeport, has a population of 62,105 people and contains two universities, both of which reopened for in-person education in mid-August. The university communities experienced a surge in cases during September to October after students returned (*61*). Students had access to frequent COVID-19 testing, and test coverage in this community was likely higher than in the general population, so infections among students might have been more likely to be reported to public health authorities. Contact rates in both Fairfield and the adjacent city of Bridgeport increased (Figs. 3 and 4) during September shortly after students arrived on campus. The consequence of this increase in contact rate is evident in the rise in case counts for Fairfield 2 to 3 weeks later.

The eastern part of Connecticut was largely spared in the first wave of infections during March to April, but Norwich (population 39,136) and nearby towns experienced a strong surge in cases beginning in mid-September (*41*, *42*). Contact rose more quickly in these towns, compared to the western part of the state, following the beginning of phase 1 in May 2020. Low testing coverage during the spring and summer of 2020, imported infections from neighboring Rhode Island, and lower compliance with social distancing measures might have played a role in outbreaks in the eastern part of the state.

Contact data do not explain all variations in confirmed noncongregate COVID-19 case counts. Although the model fits cases well overall in large cities, it can fail to capture variation in case counts in smaller cities where testing coverage is lower or in settings where case-finding effort varied over the time. For example, high case counts corresponding to outbreak investigations involving extensive testing in Danbury during August and Norwich during September/October do not directly reflect changes in contact and are not captured by the model projections.

### Contact may provide advance warning of COVID-19 cases

To assess the relationship between close interpersonal contact and COVID-19 cases without SEIR-type model assumptions about the dynamics of transmission, we fit a hierarchical Bayesian space-time statistical model to predict cases using town-level contact data. In a model that included 28 prior days of contact data, lagged contact from 3 to 7 days prior is significantly associated with current-day cases, in agreement with known features of the time to development of symptomatic disease (*62*–*67*). An increase of 10 contacts in each of the previous 28 days within an average town gives rise to an increase in cases by a factor of 1.29 (95% credible interval: [1.22, 1.37]) within that town. A model that includes contact predicts cases better than one without contact, according to goodness-of-fit criteria (*68*, *69*). The model structure and results are described in detail in the Supplementary Materials.

## DISCUSSION

Public health decision-makers track the COVID-19 pandemic using metrics—syndromic surveillance data, cases, hospitalizations, and deaths—that lag disease transmission by days or weeks. Here, we have described a method for population-level surveillance of close interpersonal contact, the primary route for person-to-person transmission of SARS-CoV-2, by using anonymized mobile device geolocation data. The contact rate can reveal high-contact conditions likely to spawn local outbreaks or areas where residents experience high contact rates, days or weeks before the resulting cases are detected by public health authorities through testing, traditional case investigation, and contact tracing. Because mobile device geolocation data are passively collected, contact rates are invariant to allocation and availability of public health resources for case finding. For this reason, contact rates could serve as a better early-warning signal for outbreaks than cases alone, especially when test volume is low. Contact rates could also have advantages over surveillance approaches using mobility metrics because interpersonal contact within 6 feet is more directly related to the likelihood of disease transmission by direct contact or respiratory droplets.

Contact rates could benefit public health efforts to prevent transmission of SARS-CoV-2 in two ways. First, community engagement programs could be directed to locations where the contact rate is high to improve social distancing practices or provide additional protective measures such as ensuring adequate ventilation, environmental cleaning, and mask use. Second, enhanced testing in areas with high contact rates and residential areas of people experiencing that contact could lead to earlier and more complete detection of cases. Earlier and more complete detection of cases enables faster and more complete isolation of cases and quarantine of contacts, which are crucial to stop transmission and stop outbreaks.

We have focused in this study on the U.S. state of Connecticut, but the usefulness of anonymized and passively collected contact data could be generalized to other settings. In the United States, where mobile phone usage is high, states or towns can implement contact surveillance at low cost by working with private sector mobile device data providers. Similar to Connecticut, other states and countries experienced constrained testing availability in the early stages of the pandemic and uneven geographic distribution of testing after test volume increased. Nonpharmaceutical interventions such as stay-at-home mandates, business and school closures, and social distancing guidelines also had uneven adoption and compliance varied across time and geography. Surveillance of contact rates could help officials better distribute testing resources and monitor intervention compliance in numerous settings. Internationally, mobile phone ownership has grown quickly but might be low in some developing countries (*70*), making contact surveillance less feasible in these settings.

The contact rate used here has several advantages over existing mobility metrics and measures of mobile device density and proximity. First, the contact rate has been designed specifically to measure interpersonal contact within 6 feet relevant to COVID-19 transmission, as defined by CDC (*1*). In contrast, mobility metrics primarily measure movement, which might not be a good proxy measure of close interpersonal contact. For each potential contact event between two devices, we use the reported device locations and horizontal uncertainty measurements to compute the probability that the devices were within 6 feet of one another. In this way, each potential contact event is weighted by the likelihood that the people carrying the devices were close enough for transmission to occur. In contrast, Unacast’s human encounters metric measures the frequency of two devices being within 50 m of one another. Because the Unacast definition includes interactions that are at a distance much farther than 6 feet, many are unlikely to involve the potential for disease transmission. The contact rate used here incorporates close interpersonal contact occurring in every location in Connecticut, not only at preselected venues [e.g., (*26*, *71*)]. For these reasons, the contact rate might be a better proxy for population-level transmission risk when there are prevalent infections.

The contact rate developed here may be a useful input to mathematical models of infectious disease transmission for prediction of COVID-19 infections or cases. In the early stages of the COVID-19 pandemic, researchers used variations of the classical SEIR epidemic model (*72*, *73*) to predict the initial wave of infections, estimate parameters such as the basic reproduction number, and assess the effects of nonpharmaceutical interventions [e.g., (*47*, *74*), in Connecticut]. These models often assumed a constant population-level contact rate that is subsumed into a transmissibility parameter or estimated contact rate from survey data collected before the pandemic (*75*, *76*). Here, we have shown that when used as an input into an epidemiological model of transmission, the contact rate gives a better fit of model projections to observed epidemiological data than other available mobility metrics.

Contact data derived from mobile device geolocation data have limitations. First, not all devices in Connecticut appear in the sample: during 1 May to 28 November 2020, we observed a total of 788,842 unique device IDs, representing roughly 22% of the 3.6 million residents of Connecticut. An analysis in the Supplementary Materials shows that there is no evidence of systematic undercoverage of mobile devices as a function of town population sizes, but coverage declines slightly in towns with higher percent of residents identifying themselves as non-White, lacking a high school degree, and below the poverty level. Undercoverage among particularly vulnerable populations could result in undercounting of potential transmission events likely to affect these populations. Second, horizontal uncertainty varies by device and location, making close interpersonal contact that occurs in some areas more difficult to detect with certainty. Third, the duration of time a device was stationary is unknown because location data are reported asynchronously and at irregular intervals. Fourth, using anonymized mobile device geolocation data, we do not observe individual-level demographic information, whether a potential contact occurred indoors or outdoors, or additional individual-level infection risk factors or risk mitigation behavior such as mask wearing, handwashing, avoidance of touching surfaces, or avoidance of crowded indoor spaces. However, CDC recommends that the determination of close contact should be made “irrespective of whether the person with COVID-19 or the contact was wearing a mask” (*77*).

The contact rate might not detect all types of close interpersonal contact relevant for disease transmission and does not distinguish between physical contact and close proximity. We exclude contact occurring at primary dwell locations, so contact between pairs of people while at their shared same primary dwell locations is not represented in the contact rate. As a result, our model projections may not adequately capture household transmission. Close contact that occurs while traveling, for example, on a bus or train, might not be detected because devices are not stationary. Devices located on different floors of the same building might report nearby locations, even if the devices are separated by one or more floors. Location information might be reported by each device at irregular intervals, so we might not observe some kinds of fleeting contact. Contact that occurs outside of Connecticut is not recorded in our dataset. In particular, we did not observe information about contact for people who live in Connecticut and work in the New York City area.

Statewide contact rates help explain Connecticut’s success in avoiding a broad resurgence in COVID-19 cases during June to August 2020, emergence of localized outbreaks during late August to September, and a broad statewide resurgence during October to December. In addition to explaining historical patterns of transmission, incorporating contact rates into an SEIR transmission model might improve prediction of future COVID-19 cases and outbreaks at the town level, which could inform targeted allocation of public health prevention measures, such as SARS-CoV-2 testing and contact tracing with subsequent isolation or quarantine. Contact rates estimated from mobile device geolocation data can help improve population-level surveillance of close interpersonal contact and guide public health messaging campaigns to encourage social distancing and in allocation of testing resources to detect or prevent emerging local outbreaks.

## Supplementary Materials

### This PDF file includes:

Supplementary Materials and Methods Supplementary Text Tables S1 to S4 Figs. S1 to S34 References
